# Tlr2 Deficiency is Associated with Enhanced Elements of Neuronal Repair and Caspase 3 Activation Following Brain Ischemia

**DOI:** 10.1038/s41598-019-39541-3

**Published:** 2019-02-26

**Authors:** Dunja Gorup, Siniša Škokić, Jasna Kriz, Srećko Gajović

**Affiliations:** 1 Croatian Institute for Brain Research, University of Zagreb School of Medicine, Šalata 12, Zagreb, HR-10000 Croatia; 2 Department of Psychiatry and Neuroscience, Faculty of Medicine Laval University, CERVO Brain Research Center, 2601, de la Canardière, Québec (QC), G1J 2G3 Canada

## Abstract

The aim of this study was to apply multimodal *in vivo* imaging to assess the influence of altered innate immunity on brain repair after ischemic lesion. Tlr2-deficient mice were compared to wild type controls, as they lack Tlr2-mediated pro-inflammatory signaling triggered by postischemic necrosis. The ischemic lesion was induced by transient middle cerebral artery occlusion for 60 min, followed by brain imaging and analysis at four time points until 28 days after ischemia. Multimodal *in vivo* imaging involved a combination of 3 modalities: (1) magnetic resonance imaging by T2-weighted scans to assess brain lesion size, (2) bioluminescence imaging of Gap43-luc/gfp transgenic mice to visualize the axonal remodeling, and (3) caged-luciferin bioluminescence imaging of DEVD-luciferin allowing for visualization of caspase 3 and 7 activity in Gap43-luc/gfp mice. This enabled innovative correlation of the MRI-determined lesion size to photon fluxes obtained by bioluminescence imaging. Our data revealed that following ischemia, Tlr2-deficient mice had higher Gap43 expression and higher levels of caspases 3 and 7 activity, which was accompanied by enhanced levels of synaptic plasticity markers DLG4 and synaptophysin when compared to wild type controls. Altered inflammation in Tlr2-deficient mice was accompanied by enhanced elements of post-stroke repair, in particular during the chronic phase of recovery, but also with delayed final consolidation of the brain lesion.

## Introduction

The therapy of stroke patients has been considerably improved since introducing thrombolysis and thrombectomy, as well as applying the stroke unit treatment^1,2^. Nonetheless, specific therapies which would address the long-term consequences of stroke are not yet available. The main reason for the lack of specific therapies is the vast complexity of interconnected events following stroke and their progression with time. This is combined with challenges of long-term follow-up in preclinical models using laboratory animals^3^. Thus, both the complexity of stroke and the improvement of animal models need to be addressed to design relevant preclinical approaches. One such approach, which was applied in the present study, is to follow the consequences of brain lesions through time with the use of *in vivo* imaging, allowing the same group of animals to be evaluated at different time points.

The transient medial cerebral artery occlusion (tMCAO) is used as an animal model for human ischemic stroke. This model involves the removal of an inserted filament following 60 minutes of occlusion, which after ischemia, allows for reperfusion of the affected territory of the medial cerebral artery. As this model combines ischemia with subsequent reperfusion, it could be of particular relevance for patients treated by thrombolysis and thrombectomy.

The aim of this study was to analyze the effects of altered innate immunity on an ischemic lesion in the mouse brain, with a specific emphasis on the aspects of neuronal stress and repair. The multimodal *in vivo* imaging adopted in this study allowed for longitudinal monitoring of animals for as long as 1 month after the lesion. As a model of reduced neuroinflammation, Tlr2-deficient mice were used since previous studies have demonstrated the reduced microglial activation and proliferation after ischemic lesion^4^.

Toll-like receptors (TLRs) are the main mediators of aseptically triggered neuroinflammation^5–8^. Necrosis following ischemia results in the release of danger/damage associated molecular patterns (DAMPs), which are then recognized by TLRs. As TLRs are expressed on the CNS resident microglia, TLR activation triggers the subsequent activation of microglia and thus an increase in the transcription of inflammatory cytokines (IFN-β, IFN-α, IL-1β i IL-6 via NFkB)^5,7,9^. As a member of the TLR family, activation of the TLR2 pathway has be shown to specifically contribute to microglial proliferation, astrocyte accumulation and recruitment of monocytes/macrophages from the peripheral circulation^4,7,10^. Tlr2 deficiency results in a reduction of the amount of Insulin like growth factor 1 (IGF-1) and Monocyte chemotactic protein 1 (MCP-1), which consequently reduces the number of activated resident microglia, as well as decreases the infiltration of CD45^high^/CD11b^+^ cells following ischemia^9^. Previous studies of brain ischemia using Tlr2-deficient mice, including our own, have shown that altering neuroinflammatory responses did not result in either beneficial or harmful consequences with regards to the lesion size, but was in fact a combination of both depending on the time or phase following ischemia^5,9^. In the acute phase, Tlr2 deficiency reduces the volume of the ischemic lesion, however in the later phase, modified inflammation associated with Tlr2 deficiency leads to delayed apoptosis and a larger sized ischemic lesion at later time points compared to the wild type (WT) animals^4^. Altered dynamics of apoptosis can be monitored through the activation of its hallmark cleaving enzyme caspase 3 (CASP3) that have been shown to rapidly increase during early postischemic responses^11^. Interestingly, a non-apoptotic role for CASP3 in controlling neuronal cytoskeleton components such as actin, MAP2, GAP43, Dbn1 and calmodulin has also been elucidated more recently^12^. Thus, CASP3 activity can be a useful representative indicator of postischemic stress^13^.

To clarify the combination of beneficial and harmful events related to modulated neuroinflammation due to Tlr2 deficiency, this study monitored ischemic brain lesion and its consequences through time by multimodal *in vivo* imaging combining magnetic resonance imaging (MRI), bioluminescence imaging (BLI) and caged-luciferin-BLI. MRI modality measured the extent of the stroke using a T2-weighted anatomical scan. BLI, based on collecting light released from transgenic animals carrying luciferase reporters when injected with luciferin, was used to monitor the activity of Gap43 promoter (Gap43-luc/gfp transgenic mouse) as a marker of axonal repair and outgrowth to assess post-stroke axonal remodeling^14^. Caged-luciferin-BLI is based on the application of non-active forms of luciferin. They liberate luciferin (i.e. as from the cage) through the cleavage by enzymes, such as caspases. In this study we applied DEVD-aminoluciferin (VivoGlo, Sigma), which after cleavage by caspases 3 and 7 (CASP3/7) liberates free luciferin from the DEVD part, enabling it to react with luciferase located in the cells of the transgenic animals. As we used Gap43-luc/gfp animals, the signal corresponded to the subset of cells expressing Gap43, which at the same time had CASP3/7 activity. Visualizing the cells exhibiting a combination of GAP43 and CASP3/7 activities represents a measure of neuronal stress and apoptosis of neurons affected by ischemia^13^.

For this particular study, several mouse lines were created and used. To achieve promoter-dependent luciferase activity in Tlr2-deficient mice, it was necessary to crossbreed the Gap43-luc/gfp transgenic animals with mice with loss of function of Tlr2. Moreover, to allow optical imaging of light we used albino variants of C57Bl/6 mice. As a result a novel transgenic mouse line Gap43/Tlr2−/− (B6-Tyr^c-Brd^- Tg(Gap43-luc/gfp)- Tlr2^tm1Kir^/Gaj) was created and specifically applied for this study. Subsequently, multimodal *in vivo* imaging of these mouse lines provided simultaneous insight into the evolution of the lesion size, neuronal stress, axonal outgrowth and apoptosis. The imaging was complemented by functional tests (weight monitoring, neurological scoring, accelerating rotarod, Y-maze, bilateral tactile stimulation (sticky tape) test, and Schallert’s cylinder test), and *ex vivo* analysis of synaptic markers (DLG4, Synaptophysin) by Western Blot.

Using the long-term follow up and standardization of BLI according to the size of ischemic lesion, as measured by MRI, we showed that in Tlr2-deficient mice a significant increase in Gap43 and caspase3/7 activity occurred in particular during the chronic phase of the brain recovery.

## Results

### Lower mortality in Tlr2-deficient mice after tMCAO

**Figure 1:**
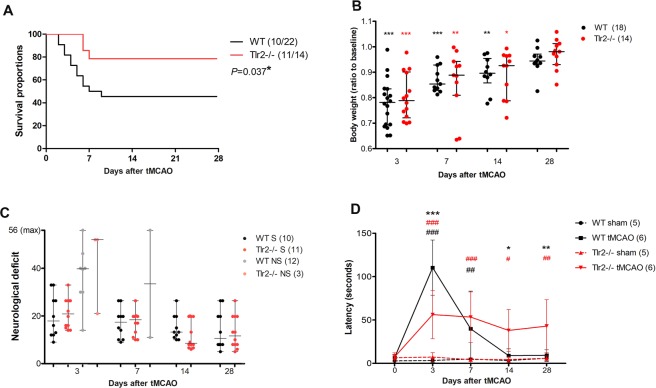
(**A**) Tlr2−/− mice survival after ischemic lesion is better than of their wild type (WT) counterparts analyzed by Log-rank (Mantel-Cox) test P = 0.037*; (**B**) Both, Tlr2−/− and WT lose body weight after ischemic lesion, and subsequently regain it after 28 days; (**C**) Mice that reached humane endpoint (grey and pale red) (non-survivors, NS) had worse neurological scores than their survivor counterparts. There was no significant difference between wild type (WT), and Tlr2−/− mice. (**D**) Bilateral tactile stimulation test revealed decreased latency in sticky tape removal from the contralateral forepaw in acute time points in Tlr2−/− mice compared to their wild type (WT) counterparts, but increased time of reaction in the later time points of 14 and 28 days. ***,** and *marking P < 0.001, P < 0.01, P < 0.05 for differences between WT controls and Tlr2−/− controls, respectively. #Are used to mark differences within the same genotype compared to their sham operated controls (WT controls vs. WT tMCAO, Tlr2−/− controls vs. Tlr2−/− tMCAO).

To compare the consequences of ischemic lesion on WT and Tlr2−/− mice, the survival after tMCAO was documented. The survival analysis showed that more Tlr2−/− mice survived than WT mice (Fig. 1A). Chances of survival of Tlr2−/− mice analysed by Log-rank (Mantel-Cox) test was 2.61 fold higher (P = 0.028) than those of WT animals. Mice (n = 36) were monitored throughout the 28 days post ischemia, with the majority of non-survivors lost during the first week (14 mice of total 15 non-survivors died during the first week).

To compare functional consequences of ischemic lesion between Tlr2−/− and WT mice, a battery of behavioural tests were used together with body weight measurements (Fig. 1). The data obtained from non-survivors were also included in order to identify putative prognostic factors that are impaired in WT compared to Tlr2−/− mice (Fig. 1C). The body weight analysis showed a significant decrease in all mice (22% loss from the baseline values) 3 days after tMCAO (Fig. 1B). Until day 14, body weight values remained significantly below the baseline, but at day 28, all groups regained their starting body weights. This parameter did not significantly differ among Tlr2-deficient and WT animals, nor did the weight loss allow for prediction of survival.

Regarding neurological scoring, there was no significant difference between Tlr2−/− and WT mice, nor between the survivors and non-survivors. Outliers with high neurological deficits belonged to the non-survivors, as the behavioural tests are similar to the assessment necessary to determine ethical humane endpoint^15^. Nevertheless, some of the non-survivors died in spite of having favourable neurological scores (Fig. 1C). Due to the nonspecific nature of neurological scoring that is suitable for observing acute behavioural deficits after tMCAO, further battery of more sensitive tests addressing sensorimotor recovery, spatial memory and exploring activities was applied^16^.

Out of four additional behavioural tests (accelerating rotarod, Y-maze, Schallert’s cylinder, bilateral tactile stimulation), significant differences between WT and Tlr2−/− mice were detected only during bilateral tactile stimulation testing using sticky tape on the contralateral forepaws. For the bilateral tactile stimulation test, latency after contact with sticky tape until the complete removal of both forepaws was noted. In WT mice, the delayed reactions of the contralateral forepaw resolved with animal recovery, while in Tlr2−/− mice, which initially fared better than WTs (latency was shorter), the delayed latency did not improve with time, and it persisted until the end of experiment (Fig. 1D). Even though Tlr2−/− mice demonstrated better survival, their functional status as measured using behavioural tests was not significantly more impaired compared to their WT controls. This was partly due to the larger proportion of deaths in the WT group where only the animals with slight impairment survived. Below the significance threshold, in Tlr2−/− mice we observed less movements in Schallert’s cylinder and lower endurance on accelerating rotarod.

### Combining MRI and BLI as a multimodal approach showed stronger Gap43 expression and CASP3 activity after tMCAO in Tlr2-deficient mice

**Figure 2:**
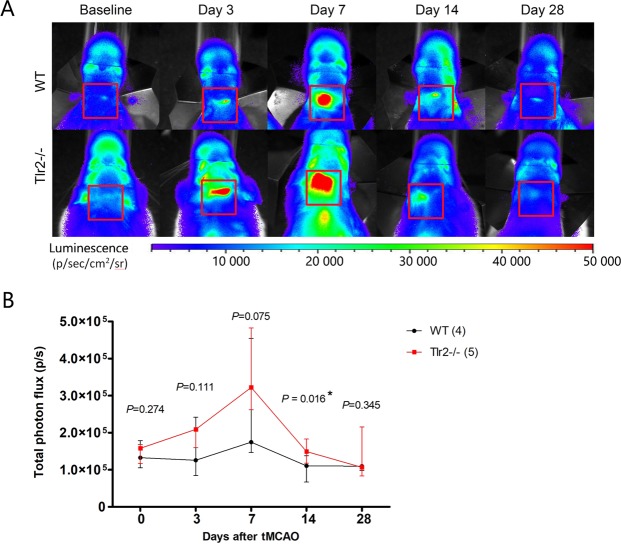
(**A**) Representative images of bioluminescence signal imaging using d-aminoluciferin for Gap43-luc-gfp (WT), and Gap43-luc-gfp-Tlr2−/− (Tlr2−/−) mice. (**B**) Total photon flux measured for Gap43-luc-gfp (WT), and Gap43-luc-gfp-Tlr2−/− (Tlr2−/−) mice showing median and interquartile range.

To detect Gap43 expression and CASP3/7 activity *in vivo*, bioluminescence imaging was performed using free D-aminoluciferin and caged-DEVD-luciferin respectively. In terms of Gap43 expression, photon flux measured in Gap43-luc/gfp transgenic mice, Tlr2-deficient mice and WT controls showed that at baseline, both mouse groups had the same values of expression. After ischemic lesion the expression increased, peaking at day 7, and returned to its baseline values at day 28 (Fig. 2).

The bioluminescent signal mirroring Gap43 expression was significantly higher (*P* = 0.016) in Tlr2−/− mice compared to WT animals at day 14. Due to large variability of the measured values, there was no statistically significant difference among the groups of the different genotype at other days, in particular at day 7 (Fig. 2B). These data led us to propose a new, ad-hoc hypothesis that the size of the lesion influenced the signal measured by BLI, i.e. bigger lesion produced more photons as measured using BLI. This hypothesis was immediately tested using MRI data on the ischemic lesion size as they were obtained, in parallel. Ischemic lesion was visualized by T2-weighted anatomical scans, with the help of T2-maps to distinguish tissue changes in the ischemic region vs. surrounding areas.

**Figure 3:**
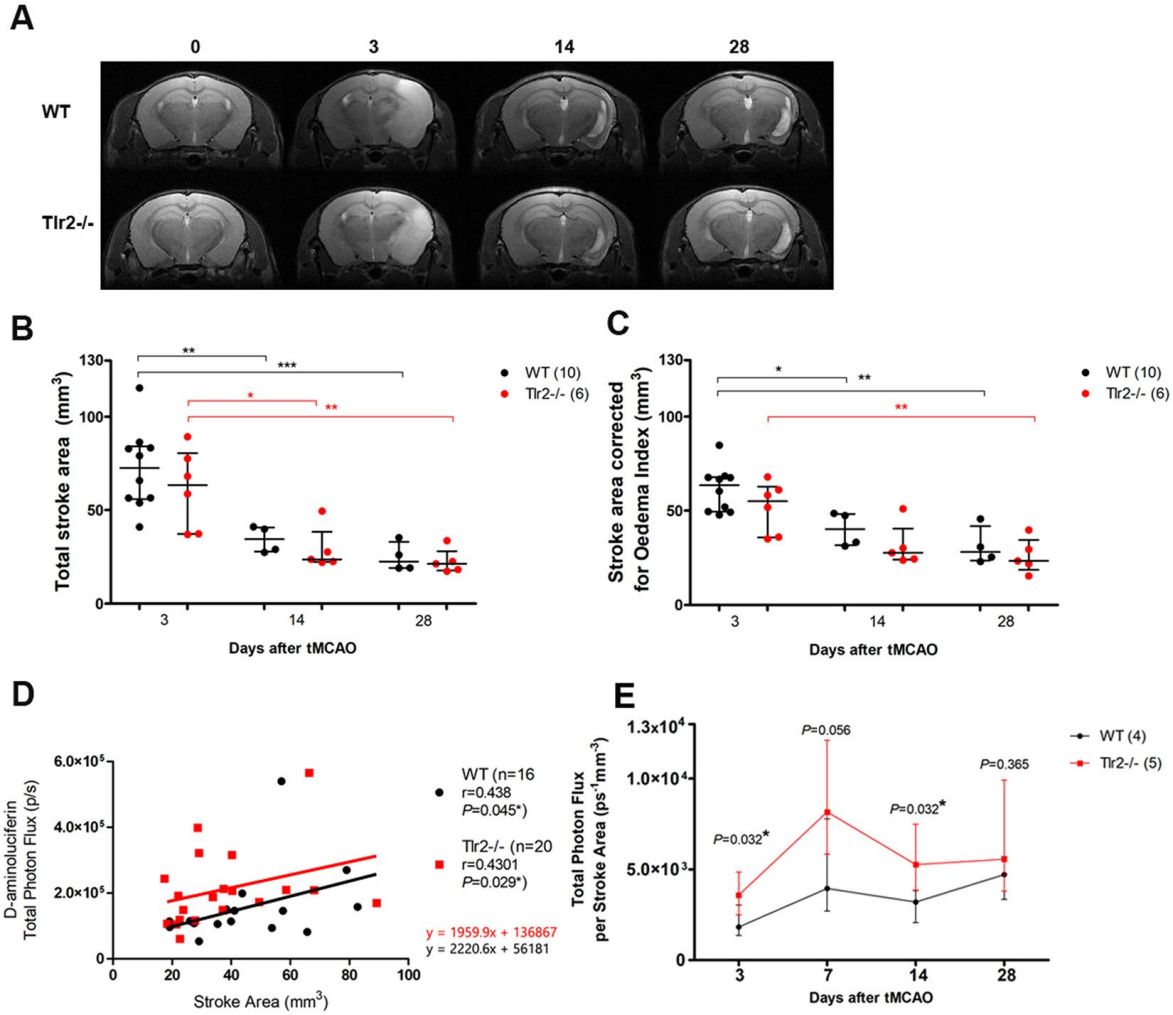
(**A**) Representative MRI T2 maps of ishemic lesion in WT and Tlr2−/− mice. (**B**) Total stroke area measured by a blinded rater shows gradual decrease in ischemic volume with no differences between the WT and Tlr2−/− mice. (**C**) Stroke area adjusted for oedema index shows the volume of ischemic lesion used for further calculation of correlations. (**D**) Correlations of BLI signal using d-aminoluciferin and Stroke area by genotype. (**E**) Total Photon Flux adjusted per stroke Area measured by MRI shows increased BLI signal in Tlr2−/− mice compared to their WT counterparts in acute and chronic time point (3 and 14 days).

MRI data was used to compare brain size between Tlr2−/− and WT mice, with results showing that brain sizes measured at baseline did not differ between the groups (data not shown). Moreover, there was no significant difference in lesion size between Tlr2−/− and WT mice at any of the time points examined (Fig. 3). Stroke area was the largest at 3 days after tMCAO and gradually decreased in size similarly in both groups (n_WT_ = 4; n_Tlr2−/−_ = 5, imaged at all time points, but the data from non-survivors were included in the analysis as well). These data do not corroborate with our previous results obtained by Nissl staining from a separate study, most likely, as the number of animals imaged was too low to show the differences^9^.

MRI data on the lesion size at each time point from the present study was further used to correlate with BLI values of Gap43 expression. The correlation was tested and proven to be statistically significant both in Tlr2−/− and WT mice (r = 0.438, *P* = 0.045* for WT, r = 0.430, *P* = 0.029* for Trl2−/−) (Fig. 3D). This confirmed our ad-hoc hypothesis and allowed to normalise the photon flux obtained by BLI to the respective lesion size obtained by MRI. The time curves of Gap43 expression normalised to lesion size differed clearly by this approach, with Gap43 expression in Tlr2-deficient mice being significantly higher at 3 and 14 days post tMCAO when compared to WT mice, and at 7 days being borderline significantly different (*P* = 0.056). Gap43 expression between the two groups was comparable only at day 28 (Fig. 3E).

**Figure 4:**
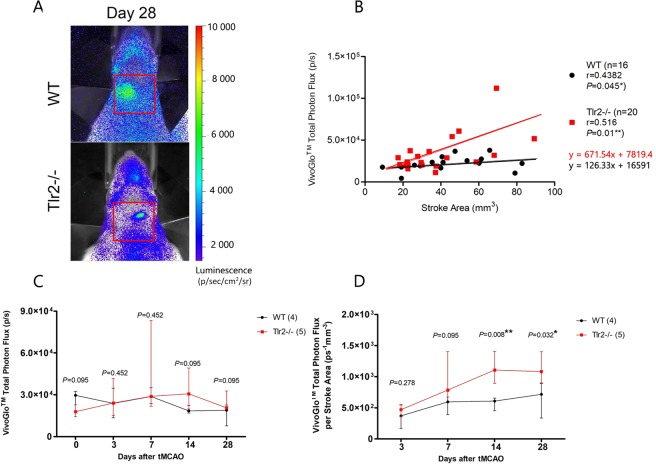
(**A**) BLI imaging after VivoGlo^TM^ injection of representative Gap43-luc-gfp (WT), and Gap43-luc-gfp-Tlr2−/− (Tlr2−/−) mice; (**B**) VivoGlo^TM^ total photon flux in correlation with Stroke Area measured by MRI. (**C**) VivoGlo^TM^ total photon flux dynamics (**D**) VivoGlo^TM^ total photon flux adjusted to Stroke area measured by MRI indicating statistically significant increase of BLI signal for Tlr2−/− mice at Days 14 and 28.

An analogous strategy was applied for caged-luciferin BLI in order to compare CASP3/7 activity between Tlr2−/− and WT mice (Fig. 4). Firstly, the original data obtained by caged-luciferin BLI were compared between groups, which showed comparable results without visible differences. Moreover, the curves crossing each other (Fig. 4C). Secondly, the correlation between lesion size and BLI-obtained photon flux was verified. Indeed, the correlation was present for both groups (r = 0.438, P = 0.045* for WT, r = 0.430, P = 0.029* for Trl2−/−; Fig. 4B). Finally, the BLI photon flux was normalised according to the lesion size and the time curves of the two groups were clearly distinguishable, showing in Tlr2-deficient mice higher CASP3/7 activity in Gap43-expressing cells, in particular in the chronic stage of recovery, where the activity was higher at days 14 and 28 (*P* = 0.008, *P* = 0.032, respectively).

In conclusion, the multimodal approach of combining MRI and BLI resulted in the observation of significant differences between the analysed groups, convincingly demonstrating the influence of reduced inflammation on Gap-43 expression and CASP3 activity as exhibited in Tlr2-deficient mice.

### Higher GAP43 and CASP3 in Tlr2-deficient mice were accompanied by increased expression of synaptic markers

**Figure 5:**
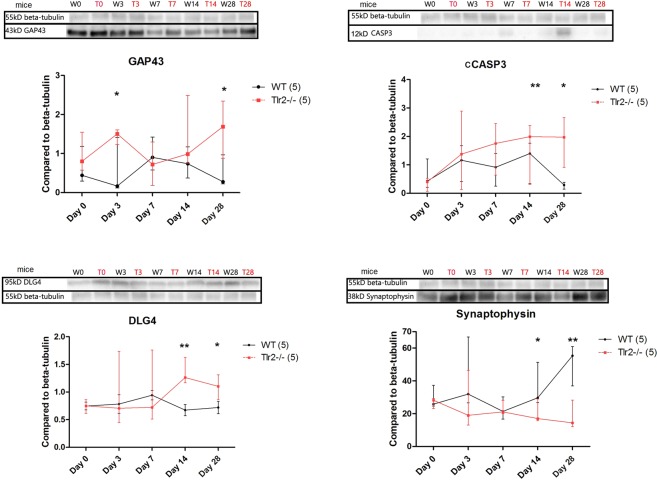
Western Blot with analysis per time point of WT and TLR−/− showing median and interquartile ranges with statistically significant differences at chronic time points for GAP43 and CASP3, as well as for synaptic markers of DLG4 and synaptophysin. ***,** and *marking *P* < 0.001, *P* < 0.01, *P* < 0.05 for differences between WT controls and Tlr2−/− controls, respectively. The blots shown here were cut from the bigger gel, therefore boxed here, and the full-length blots were provided in Figure S1.

To confirm bioluminescence findings, protein levels of GAP43 and CASP3 in the brain of mice with ischemic lesion were determined using Western Blot. This was supplemented with the measurement of the expression of synaptic markers DLG4 and synaptophysin, indicating overall quantity of synapses, as a direct measure of synaptic recovery (Figs 5 and S1).

Levels of GAP43 in WT mice reached its maximum at day 7 post ischemia and then decreased to baseline levels at day 28, in conformity with previous studies^14,17^. In Tlr2−/− mice, GAP43 values at day 28 were significantly higher than at baseline, contrary to the decline as demonstrated in WT mice. Interestingly, GAP43 levels followed the dynamics of synaptophysin (Fig. 5D), indicating the connection between axonal outgrowth (as measured by GAP43) and synaptic plasticity (as measured by synaptic markers) after tMCAO.

CASP3 quantities rose in both groups, with significantly higher values measured in Tlr2−/− mice when compared to WT animals. At day 28, CASP3 resolved to baseline values in WT mice, while in Tlr2−/− mice CASP3 activity persisted at significantly higher levels (Fig. 5D).

Western blot analysis of the synaptic proteins DLG4 and synaptophysin showed that the baseline values of both were similar in both animal groups, but after tMCAO, DLG4 increased in the Tlr2−/− mice at day 14 and remained elevated until day 28, when synaptophysin increased as well. At day 28, both of these proteins were expressed at lower levels in the WT group when compared to Tlr2−/− mice. In conclusion, Tlr2-deficiency led to a slower yet higher increase in the expression of GAP43 and synaptic markers, with longer persistence of high CASP3 levels than in WT mice.

## Discussion

The MCAO stroke model remains the golden standard for bridging the translational gap between basic research and clinical applications in spite of inherent variability^18^. The advantage of the use of modern tools such as multimodal imaging is the ability to counteract variability and provide standardization. In this study, multimodal imaging was applied in the analysis of the ischemic brain lesion using MRI, BLI and caged-luciferin BLI. The multimodal approach allowed for the normalization of the values of BLI to the size of the ischemic lesion obtained by MRI. This provided the analytical power to achieve significant differences between tested (Tlr2-deficient) and control groups (WT animals) using a rather small number of animals (n = 5 per group), which in the case of using a single modality, would remain a highly underpowered study. This is particularly evident in the case of caged-luciferin-BLI, which as a single modality did not reveal any differences, however when adjusted to the lesion size, demonstrated differences that were clearly pronounced and statistically significant.

Subsequently, the major outcome of this study was establishing a new way of combining MRI and BLI, based on the correlation of the MRI-determined lesion size with the BLI and caged-luciferin BLI photon flux obtained at the same time point. This innovative approach was applied to measure Gap43 expression and CASP3/7 activity, and correlation with the ischemic lesion size was confirmed for both markers. This showed that the combination of MRI and BLI could be used for the transgenic reporters, where promoter of interest controls luciferase expression, as for the enzymatic approach, where the enzyme of interest cleaves the caged luciferin and releases its free form. Nonetheless, we cannot claim that this approach would work for any bioluminescent *in vivo* marker, as the correlation between modalities would need to be firstly verified and this would then indicate if a multimodal approach could be more informative than using a single imaging modality.

When discussing the clinical relevance of MCAO to the ischemic stroke, the variability of the lesion size is similar in mice and humans. Hence, if we could overcome the issue of lesion variability in a constructive way, namely, by measuring the deviation of the every animal, then the study design would provide the answers to experimental questions without increasing the number of animals used. This would potentially enhance the clinical relevance of the MCAO model in preclinical studies of ischemic stroke. Our results in the present study suggest that multimodal imaging may contribute in this direction of overcoming lesion variability and that information on lesion size obtained by MRI can be informative for other imaging modalities (and eventually other type of measurements). Moreover, this endorses the reinforcement of regulatory bodies requesting results with limited number of animals^19^. It is important not to jeopardize studies by reducing the number of animals used, and our multimodal imaging approach in the present study provides a valuable alternative.

Neuroinflammation resulting from ischemia reperfusion injury was altered in Tlr2-deficient mice when compared to WT mice. Several markers measured in the present study were upregulated in a time-dependent manner, namely, GAP43, CASP3/7, DLG4 and synaptophysin. Gap43 expression was increased in Tlr2-deficient mice compared to WT controls, suggesting an augmented attempt of neural cells to rearrange their axons. Removing damaged neurons by apoptosis is another way of repair shown by CASP3/7 activity. In this study, this visualization of apoptosis was not wide-ranging, as only a subset of Gap43-luc-expressing cells was available for imaging. While caspase-7 is expressed on microglia during neuroinflammation, caspase-3 is a more dominant effector of the caspase cascade in neurons^20,21^. Therefore the signal harvested from GAP43-luc-gfp mice can be attributed predominantly to the cleaved caspase-3.

Caged-luciferin BLI using DEVD-aminoluciferin was previously shown as feasible approach in measuring caspases after ischemic lesion. The co-localization of GAP43 and CASP3 is not only specific for the visualization of apoptotic cells, but it also corresponds to neurons in stress^13^. When caged-luciferin BLI was compared to Western blot results of CASP3, Tlr2-deficient mice had higher protein levels than the WT controls, but the time-curves were not exactly the same. The observed difference can be caused by the fact that Western blot measured total brain CASP3, while caged-luciferase BLI measured only the subset present in Gap43 cells, where the signal decreased in the late time points. Taking into account the intracellular roles of CASP3 (apoptosis, pruning) and GAP43 (sprouting), the caged-luciferase BLI signal may be interpreted as the amount of neuronal stress in the early phase after ischemic lesion, and consolidation and repair in the later phase. This was confirmed using the markers of synaptic plasticity (DLG4, synaptophysin), which increased at the chronic time points in Tlr2-deficient mice in comparison to WT mice. Together with lower mortality in Tlr2-deficient mice, this remains in consistency with previous findings of delayed apoptosis of neurons and different evolution of repair in these mice^4,5^.

The question that remained unsolved in this study is whether the mice with an altered innate immunity response and enhanced elements of repair do indeed recover better than the WT controls. The neurological scoring and the behavioral tests did not show functional improvement, moreover, in the bilateral tactile stimulation, Tlr2-deficient mice were worse in the chronic phase than the WT controls. This controversial outcome has previously been reported where delayed apoptosis in the Tlr2-deficient mice was accompanied by the larger ischemic lesion in the chronic phase^9^. The enhanced caspase activity actually delayed the final consolidation of the lesion, and perhaps the final verdict could be made only after a longer analysis period than the 28 days assessed in the present study.

In conclusion, in the acute phase following ischemia, Tlr2-deficient mice seem to be protected from early deleterious consequences of ischemic lesion and have less mortality than their WT counterparts. In the chronic phase, they have higher expression of markers for neural repair, resulting in prolonged brain remodeling and delay of final consolidation of the lesion. The application of multimodal imaging allowed for a longitudinal study of the measured markers without the need for an increase in the number of animals used. Therefore, multimodal imaging offers a realistic possibility for the assessment of complex post-stroke events, with the potential for the evaluation of novel medical interventions (drugs, patient rehabilitation and stem cells).

## Materials and Methods

### Animals

All animal handling and surgery was approved by the Ethics Committee of the University of Zagreb, School of Medicine. All experiments were performed in accordance with relevant guidelines and regulations.

The experiments were carried out on 12–16 weeks old male mice bred at the animal facility of the Croatian Institute for Brain Research. B6N-Tyr^c-Brd^/BrdCrCrl represents the albino variant of the C57Bl/6 inbred strain. B6-Tg(Gap43-luc/gfp)/Kri generated by Kriz’s research group has luciferase and GFP reporters under the control of the Gap43 promoter^14^. B6.129-Tlr2^tm1Kir^/J line represents loss of function of Tlr2 gene^4^.

For the purpose of this study we created new mouse lines. B6-Tg(Gap43-luc/gfp) 10Kri was mated with B6N-Tyr^c-Brd^/BrdCrCrl to bring the transgenic line onto an albino background resulting with B6-Tyr^c-Brd^- Tg(Gap43-luc/gfp)/Gaj. This mouse line was then mated further with B6.129-Tlr2^tm1Kir^/J mice in order to obtain mice with loss of function of Tlr2, which also have the Gap43 transgene and are on the albino B6 background, allowing to follow Gap43 expression in the knock-out mice. The resultant mouse line was B6-Tyr^c-Brd^-Tg(Gap43-luc/gfp)- Tlr2^tm1Kir^ /Gaj.

Subsequently, in this study we compared loss of Tlr2 function (depicted as Tlr2−/−) with their no loss counterparts (depicted as WT). Both mouse lines were also Gap43 transgenics on an albino B6 background, i.e. B6-Tyr^c-Brd^- Tg(Gap43-luc/gfp)- Tlr2^tm1Kir^/Gaj (depicted as Tlr2−/−) was compared to B6-Tyr^c-Brd^- Tg(Gap43-luc/gfp)/Gaj (depicted as WT).

The animals were genotyped by polymerase chain reaction (PCR) for detection of the luciferase and Tlr2 gene as suggested by Jackson Laboratory and previously described^14,22^.

### Transient middle cerebral artery occlusion (tMCAO)

For inducing unilateral ischemic lesion of the brain, we used the tMCAO method. Mice were anaesthetised with 2% isoflurane inhalation anaesthesia. An intraluminal silicon-coated filament was introduced in the left middle cerebral artery for 1 h followed by reperfusion as previously described^23^. Sham operated animals were subjected to the anaesthesia and filament introduction/retrieval without an occlusion period.

### Behavioral tests

To compare functional recovery after tMCAO between experimental groups we used 5 different tests: neurological deficit scoring (56 points neuroscore), accelerating rotarod, Y-maze, bilateral tactile stimulation (sticky tape) test, and Schallert’s cylinder test. In order for the laboratory animals to get used to the rater, all the tests were started 2 weeks before the tMCAO and performed on a weekly basis. After the surgery, behaviour was tested on the day 1, 7, 14, 21 and 28.

### *In Vivo* Bioluminescence imaging (BLI)

GAP43 transgenic mice were anesthetized with 2% isoflurane in 100% oxygen in an induction chamber, and injected intrapretioneally (i.p.) with different luciferase substrates on consecutive days: VivoGlo (VivoGlo Caspase 3/7 Substrate; Z-DEVD-Aminoluciferin Sodium Salt, Promega, Madison, WI, USA) followed by luciferin (XenoLight D-Luciferin - K+ Salt Bioluminescent Substrate, PerkinElmer, Waltham, MA, USA), 24 hours after^4,13^. Animals were individually placed in the heated light-tight imaging chamber of IVIS SPECTRUM Imaging System (PerkinElmer, Waltham, MA, USA) with continuous inhalation anaesthesia of 2% isoflurane–oxygen mixture at 1 L/min. To obtain the baseline values, imaging was done 3 days before the surgery and then 3, 7, 14, and 28 days after tMCAO. Total flux of photons was measured using the Living Image 4.3 acquisition and imaging software (PerkinElmer, Waltham, MA, USA), as described previously^13^.

### Magnetic resonance imaging (MRI)

Animals were imaged 3 days before tMCAO and at 3, 14 and 28 days after tMCAO for establishing baseline and follow up images, respectively.

Magnetic resonance imaging was performed on a 7 T system (BioSpec 70/20 USR with Paravision 6.0.1. software version, Bruker Biospin, Germany) in a Tx/Rx configuration, using an 86 mm transmit volume coil (MT0381, Bruker Biospin, Germany) for transmitting (Tx) and a 2-element mouse brain surface receive coil (MT0042, Bruker Biospin, Germany) for receiving (Rx). The animals were placed in a supine position inside a water-heated Bruker mouse bed (Bruker, Germany), and the position of their head was secured by help of the tooth bar and ear bars.

Prior to imaging, animals were anesthetised in an induction chamber with a mixture of 70/30% N_2_/O_2_ containing 4% isoflurane (Isoflurane, Abbott, UK). During scans, anaesthesia was maintained with a delivery of 1–1.5% isoflurane in the same N_2_/O_2_ mixture, and monitored via observing the respiratory rate with an optical probe (Medres, Cologne, Germany). The respiratory rate was kept in the range 80–100 breath/min during the whole scan. Body temperature of the animal was maintained at 37 ± 0.5 °C by controlling the temperature of the water heated bed and an additional body cover with a feedback-controlled circulating heating pump and was monitored using a MR-compatible rectal temperature probe (Medres, Cologne, Germany).

The scan protocol consisted of a preparatory phase and main scans. In the preparatory phase, first a low-resolution GE pilot scan (Multi-Slice Localizer) was performed, followed by an additional TSE scan (TurboRARE) in the sagittal plane to enable precise longitudinal alignment of the slices, and an additional shimming over the region of interest using MAPSHIM sequence. The main scans included a high-resolution T2-weighted anatomical scan for measuring the extent of the stroke and a T2-map scan protocol to assess the level of tissue change in the ischemic region. T2 maps were calculated from measured data using built-in post-processing macros of Paravision software.

In all main scans, the field of view was set to 16 × 10 mm, and the slice thickness and gap between slices were 0.5 mm and 0.1 mm, respectively. The slice package extended from rhinal fissure to the anterior part of the cerebellum and was aligned, in all time points, on the second slice, which was placed on the rhinal fissure. Further scan parameters were set as follows:

#### High-resolution T2-weighted scan

Turbo Spin-Echo sequence (TurboRARE, RARE = 8), TR/TE = 3000/30 ms, Nex = 6, In-plane resolution = 90 × 90 um, total time = 4 min.

#### T2-map scan

Multi-echo Spin-Echo sequence (MSME, Necho = 15), TR/TE = 2500/(8, 16, …, 120 ms), Nex = 5, In-plane resolution = 111 × 111 um, total time = 19 min.

Total duration of the imaging protocol, including anaesthesia and positioning, was around 50 min.

Segmentation was performed by manual delineation of ipsilateral and contralateral hemisphere and ischemic lesion in ImageJ1 (NIH, Bethesda, USA) taking into account possible stroke density differences and contiguity of the lesion area throughout the MRI image stack. MRI exclusion criteria were applied during the first post-tMCAO imaging and consisted of un-apparent lesion, lesion size below 20 pixels, and haemorrhagic conversion of ischemic lesion.

### Western blot

To detect levels of proteins involved in inflammation and regeneration after brain ischemia, mice stroke affected lateral portions of the hemisphere were isolated and snap-frozen in liquid nitrogen and stored at −80 °C. Proteins were isolated by mechanical homogenisation in SDS-urea-β-mercaptoethanol solution containing proteinase inhibitor on ice. Electrophoresis and transfer on the PVDF-membrane was done using BioRad Mini-PROTEAN3 System (BioRad, Hercules, CA, USA).

Primary antibodies were β3-Tubulin (5568 S, Cell Signalling Technology, Beverly, MA, USA) as a loading control, DLG4 (PSD95) (3450, Cell Signalling Technology Beverly, MA, USA), GAP43 (AB5220, Merck, Kenilworth, NJ, USA), synaptophysin clone SY38 (MAB5258, Merck, Kenilworth, NJ, USA), and anti-cleaved CASP3 (Cell Signaling, Danvers, MA, USA). Secondary antibodies were goat anti-mouse, and goat anti-rabbit (32430 and A27011, Thermo Fisher Scientific, Waltham, MA, USA). The chemiluminiscent signal achieved by Western Lightning – EXL Enhanced Chemiluminiscence Substrate (Perkin Elmer, Waltham, MA, USA) was imaged by ChemiDoc MP System (BioRad, Hercules, CA, USA) and analysed using Image Lab Software (BioRad, Hercules, CA, USA).

### Experimental groups

For behavioural analysis 4 groups were followed: Gap43 sham (5) and Gap43Tlr2−/− sham (5) as controls, and Gap43 (6) and Gap43Tlr2−/− (6) mice that underwent the tMCAO. During the experiment, 4 Gap43 and 2 Gap43Tlr2−/− mice had an early humane endpoint (HEp) due to tMCAO, and 1 Gap43 mouse was retroactively excluded from the experiment due to haemorrhage established during the brain isolation (total 29 mice).

For Western Blot, 5 mice were used per group (Gap43 and Gap43Tlr2−/−) to determine relative protein values for the baseline (naïve controls, Day 0), and for 3,7,14 and 28 days post tMCAO (total 50 mice). There were no sham-operated animals whose tissue was isolated at each time point, due to the reduction of the number of animals, and as a control only naïve mice were used and included on the graph as Day 0.

For *in vivo* imaging 15 Gap43 animals were used, with 3 of them had no detectable lesion on MRI and therefore were removed from the experiment. Furthermore, 8 mice from that group reached the humane end point before the day 28 (Non-Survivors). Out of 9 Gap43Tlr2−/− mice enrolling the imaging, only 1 reached the humane endpoint before the day 28, and 3 animals were removed from the experiment due to the MRI exclusion criteria for the lesion identification (total 26 mice).

Total number of mice used was 103.

### Statistical analysis

Survival proportions were analysed by Log-rank (Mantel-Cox) test.

Body weight, neurological deficit scoring, behavioural analysis, MRI and BLI data were analysed by 2-way analysis of variance of 4 groups followed by Bonferronni’s multiple comparison test.

For MRI data, BLI data, and neurological deficit score correlation, graphs were assembled observing the R^2^ values of correlation coefficient, and data were analysed by comparing the 2 groups at each time point by Mann-Whitney test.

For Western blot results Mann-Whitney test was used to compare the protein quantities from the samples collected at different time points and between the different groups (WT, Tlr2−/−).

Differences were considered statistically significant with *P* < 0.05.

## Supplementary information

Supplementary Information
