# Supplementary material for: Tlr2 Deficiency is Associated with Enhanced Elements of Neuronal Repair and Caspase 3 Activation Following Brain Ischemia
Source: Sci Rep. 2019 Feb 26;9:2821. doi: 10.1038/s41598-019-39541-3 (PMC6391535; doi:10.1038/s41598-019-39541-3)
Supplement: Supplementary file 1 — Supplementary Information [file 41598_2019_39541_MOESM1_ESM.pdf]

TLR2-DEFICIENCY IS ASSOCIATED WITH ENHANCED ELEMENTS OF NEURONAL REPAIR AND CASPASE 3 ACTIVATION FOLLOWING BRAIN ISCHEMIA

Gorup Dunja[1], Siniša Škokić[1], Jasna Kriz[2], Srećko Gajović[1]*

[1]Croatian Institute for Brain Research, University of Zagreb School of Medicine, Šalata 12, Zagreb HR-10000, Croatia

[2]Department of Psychiatry and Neuroscience, Faculty of Medicine Laval University, CERVO Brain Research Center, 2601, de la Canardière, Québec (QC), G1J 2G3, Canada

*corresponding author: srecko.gajovic@hiim.hr

SUPPLEMENTARY INFORMATION:

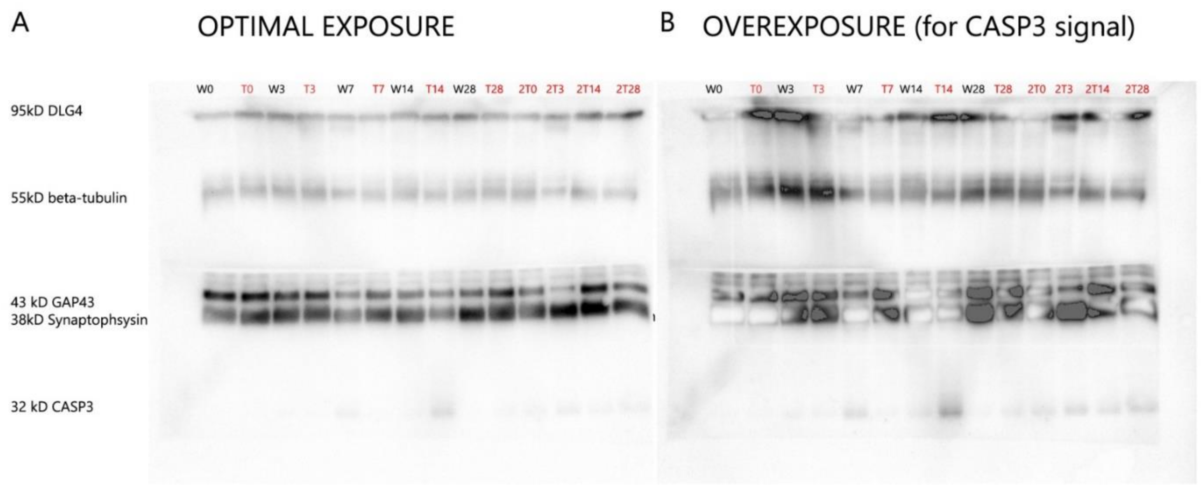

Supplemental Figure S1. Full-length Western blots used for cropped blots in the Figure 5, where each investigated proteins (DLG4, beta-tubulin, GAP43, Synaptophysin, CASP3) were displayed delineated in divided squares. Mice markings are black for WT (prefix "W"), and red for Tlr2-/- strains (prefix "T"), followed by the number of days after tMCAO (0, 3, 7, 14, 28 days). A) Optimal exposure used for the analysis of DLG4, GAP43, Synaptophysin and beta-tubulin signals. B) Overexposure used for the analysis of CASP3 signal.

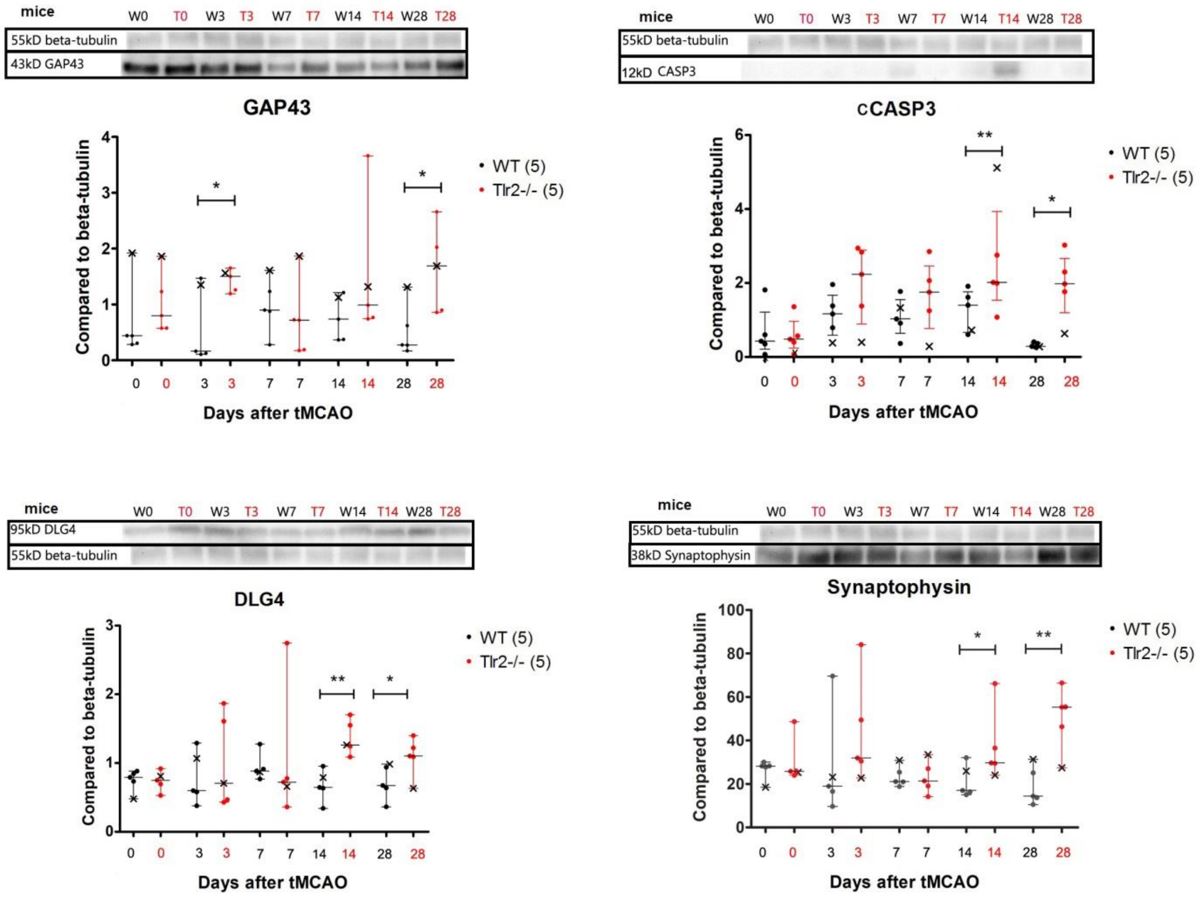

Supplemental Figure 2 Western Blot with analysis per time point of WT and TLR-/- showing median with interquartile range and statistically significant differences in Mann Whitney test at chronic time points for GAP43 and CASP3, as well as for synaptic markers of DLG4 and synaptophysin. *, ** and * marking *P*<0.001, *P*<0.01, *P*<0.05 for differences between WT controls and Tlr2-/- controls, respectively. Blotted values shown are marked by "x" on the graph, or their intraindividual means from additional blots.**
